# Development of a multiplex mass spectrometry method for simultaneous quantification of urinary proteins related to respiratory health

**DOI:** 10.1038/s41598-021-89068-9

**Published:** 2021-05-12

**Authors:** Sarah J. D. Nauwelaerts, Nancy H. C. Roosens, Alfred Bernard, Sigrid C. J. De Keersmaecker, Koen De Cremer

**Affiliations:** 1 Transversal Activities in Applied Genomics, Sciensano, Brussels, Belgium; 2 Louvain Centre for Toxicology and Applied Pharmacology, University catholique de Louvain, Woluwe, Brussels, Belgium; 3 Platform Chromatography and Mass Spectrometry, Sciensano, Brussels, Belgium

**Keywords:** Biomarkers, Environmental impact

## Abstract

Respiratory health of children is a health priority. Club cell protein (CC16) is an interesting biomarker of lung diseases and adverse effects towards the airway epithelium integrity. Osteopontin (OPN) and nuclear factor-kappa B (NF-κB) also play a role in respiratory health. The use of urine as biomarker source is useful in studies involving children but necessitates proper adjustment for physiological confounders influencing the urinary excretion, potentially characterized with beta-2-microglobulin (β2M), retinol binding protein 4 (RBP4) or myoglobin (MYO), as well as adjustment for possible renal dysfunction, characterized by human serum albumin (HSA). The simultaneous quantification of all these proteins in urine could facilitate children’s health monitoring. A multiple reaction monitoring method (MRM) was developed and validated for the relative quantification of the seven mentioned urinary proteins. A total of nine proteotypic peptides were selected and used for the relative quantification of the seven proteins. The MRM method was completely validated for all proteins and partially for OPN. LOQ’s ranged from 0.3 to 42.8 ng/ml, a good reproducibility and a good linearity were obtained across the analytical measurement range (r^2^ > 0.98). The method yielded varying correlations (r^2^ of 0.78, 0.71, 0.34 and 0.15 for CC16, β2M, RBP4 and HSA respectively) with available immunoassay data. It also allowed the identification and successful quantification of β2M and RBP4 as a protein candidate for adjustment of renal handling and dysfunction. All proteins were detected in the urine samples except for MYO and NF-κB. Our validated MRM-method is able to simultaneously quantify in urine biomarkers of airway epithelium integrity and biomarkers of variation in renal function and urinary dilution. This will allow to investigate further in future studies if urine can be used as a good surrogate source for biomarkers of airway epithelium integrity, and to understand the complex relationship between cause and effect in children’s respiratory health monitoring.

## Introduction

Respiratory health of children is among the priorities of national and international environmental health programs. Children are especially vulnerable to environmental stressors like poor air quality^1,2^. Monitoring exposure, effect and susceptibility in children’s cohorts, using biomarkers may help to understand the complex relationship between cause and effect and is also of critical importance for health care management purposes, public health decision making, and primary prevention activities^3^. In this context, the club cell protein (previously named Clara cell protein; hereafter referred to as CC16), is an interesting protein biomarker. This small protein (16 kDa) is produced by the club cells in the distal bronchioles of the lung, by the nasal epithelium cells and all along the trachea-bronchial tree^4–6^. Once secreted in the epithelial lining fluid of the respiratory tract, it leaks in small amounts across the airway epithelium into the blood. There it is rapidly cleared into the urine by glomerular filtration^5^. Although its biological function is not yet fully elucidated, several studies have shown a protective role including anti-inflammatory and immunomodulatory properties^7–10^. Therefore, CC16 has been described to be a potential interesting biomarker of lung diseases, as several studies in children and adults have associated lower serum levels of CC16 with decreased lung function and increased risks of wheezing in asthma, bronchiolitis, allergic rhinitis, lung cancer and chronic obstructive pulmonary diseases^11–16^. Moreover, CC16 is also an interesting biomarker of adverse effects towards the airway epithelium integrity. Indeed, studies have shown a rapid increase of serum CC16 when the airway epithelium is disrupted due to respiratory irritants such as ozone or other acute induced inflammatory processes^17,18^. When the exposure to a variety of irritants, including air pollutants, becomes chronic, the number of club cells in the deep lung decreases, leading to reduced serum CC16 levels^16,19^.

Other protein biomarkers were also described as potentially relevant to monitor respiratory epithelium integrity. Osteopontin (OPN) is an extracellular matrix protein and cytokine, produced by the airway epithelial cells and inflammatory cells around the airways. Studies have shown increased serum levels in children with allergic rhinitis and asthma^20,21^. NF-κB is also known to be an inflammatory biomarker of the upper airway epithelium^22,23^. Many studies indicate an enhanced NF-κB pathway activation in asthmatic tissues^24–26^ and nasal lavage fluid (NALF), where it was found to be a noninvasive marker for assessment of different grades of asthma severity^27^. However, its relation to lower airway inflammation has not been fully studied in childhood asthma.

Blood samples are the most commonly used sources for the measurement of biomarkers, but for ethical and practical reasons this is not feasible in most studies involving young children. Therefore, the use of a noninvasive biofluid such as urine, could be an interesting alternative for monitoring respiratory health in children. Indeed, urine is a valuable source of protein biomarkers and was already used for the measurement of the above mentioned CC16, OPN and NF-κB in different types of studies of respiratory health^28^ and other health conditions^29,30^. However, the main challenge when measuring low-molecular-weight (LMW, < 40 kDa) proteins such as CC16 and OPN in urine, is the proper adjustment for physiological confounders influencing the urinary concentrations, in particular the urine dilution and protein tubular reabsorption capacity. Creatinine is often used but it does not take into account the tubular reabsorption of the protein, correcting only for the dilution. The most commonly used biomarkers of the tubular reabsorption capacity of proteins are the LMW proteins retinol binding protein 4 (RBP4) and beta-2-microglobulin (β2M). These two proteins have a size close to that of CC16, but their urinary levels are on average 40 times higher than that of CC16^31,32^. Although never used as a biomarker of tubular function, myoglobin (MYO) might be of interest, as is has similarities with CC16 for both its size (16.7 kDa) and its low (a few µg/l) or even absent urinary levels in healthy subjects^33^. Additionally, when looking into urinary proteins, it is always of interest to measure human serum albumin (HSA), a high-abundance urinary protein, that may influence the tubular reabsorption of LMW proteins by competitive inhibition^34^. Additionally, HSA represents the glomerular filtration and can therefore be used as a biomarker of renal dysfunction^35^ which is relevant information when investigating urinary samples.

Investigation of protein biomarkers is often done using immunochemical assays. These assays are expensive to develop and time-demanding especially when samples are numerous and multiple proteins have to be measured consecutively, which is usually done with simplex assays. This is especially more problematic, when investigating potential adjusters for renal function, where reliable comparison and limited inter-assay variation are required. Additionally, these assays are not always commercially available for the studied proteins and cross-reactions can occur influencing the validity of the measurements^36–39^. An alternative high-throughput technology for absolute or relative quantification of proteins is proteomics using liquid chromatography coupled with tandem mass spectrometry (LC–MS/MS) in multiple reaction monitoring mode (MRM). When performing this targeted coupled technique, a triple quadrupole mass spectrometer is used to allow the simultaneous systematic acquisition of specific proteotypic peptides after tryptic digestion of the proteins in the sample. These peptides uniquely identify each selected protein and serve as a surrogate for the protein of interest, eventually leading to the absolute or relative quantification of the selected proteins in complex biofluids, such as urine, nasal lavage fluid (NALF) or blood^40^. A main advantage of this technique is that the number and identity of the proteins can be tailor-made for each project and that this is independent from the tests that are commercially available^41–43^. Apart of reducing the sample volume, the multiplex aspect also limits the variations that can occur when running each assay separately. Each variation that is seen in the levels of the various proteins is therefore more likely to reflect the true abundances within the sample.

In the present study, we aimed to develop and validate a MRM method targeting simultaneously the potential protein biomarkers for respiratory health (CC16, NF-κB and OPN) or for renal dysfunction (HSA) as well as potential adjusters of renal handling (RBP4, MYO and β2M) and to allow relative quantification in urine from a study cohort of young children. To our knowledge, this is the first study using this MRM technique for the measurement of CC16 in urine in children. Additionally, comparison with results from the classic immunochemical assays was done when these were available from previous studies involving a selection of children based on the same cohort^28^. Finally, we explored the most appropriate method to adjust urinary CC16 levels for variations of both diuresis and renal protein handling. The implementation of such an MRM tool will facilitate future studies further investigating urine as surrogate source of biomarkers of respiratory health in children.

## Methods

### Sample collection and analyses

Urine samples were collected during two time points from children, recruited in the framework of a 2-year prospective field study between 2008 and 2010. The ethics committee of the faculty of medicine of the catholic university of UCLouvain approved the study protocol (Registration number B403201734310). Research was performed according to all relevant regulations and informed consent was obtained from the parents of the participating children. More details can be found elsewhere^44,45^. These children were recruited from multiple Belgian schools, located mainly in the areas of Liege and Brussels, in the framework of an epidemiological study investigating the different effects of a selection of environmental stressors on the child’s health. Examinations of children, which took place in schools, included the measurement of body weight and height and the collection of a urine sample. During the two time points of the study, children were examined between 9:00 a.m. and 3:00 p.m., and urine samples were collected before noon and immediately stored at − 20 °C.

During this 2-year prospective study, the concentrations of CC16, RBP4, β2M and HSA in urine were measured using automated immunoassays based on the agglutination of latex particles, using Dakopatts antibodies and standards based on commercially available proteins or proteins purified in the laboratory^46–49^. Urinary creatinine was quantified with the Beckman Synchron CX5 Delta Clinical System. A total of 66 children (mean of 6.38 year old, 39 boys, mean BMI of 19.90 kg/m^3^) were randomly selected for further investigation in this study. From these children, we obtained 72 urine samples, collected during the first and/or second time point, which were further used for comparing the latex immunoassay (LIA) results with that of the MRM method. Within this selection, data for quantification in urine with LIA were missing for 3 samples for CC16, 31 samples for β2M, 2 samples for HSA, 2 samples for RBP4 and 1 sample for creatinine.

### Chemicals and reagents

The stable isotope-labeled peptides were supplied by Thermo Fischer Scientific and immediately stored at − 20 °C. The intact proteins were commercially available. Recombinant human CC16, recombinant human NF-κB p65, recombinant human OPN, recombinant human β2M, native human MYO and native human HSA were supplied by Abcam. Human RBP4 was supplied by LeeBio.

Ammonium bicarbonate (NH_4_HCO_3_), bovine serum albumin (BSA), dl-dithiothreitol (DTT), iodoacetamide (IAA), TRIZMA base, calcium chloride dihydrate and formic acid (FA) (99–100%) were supplied by Sigma-Aldrich. The lyophilized Rapigest SF was supplied by Waters. Sequencing grade modified trypsin was bought from Promega. Methanol and Acetonitrile HPLC-S Gradient grade were from Biosolve. Water was purified and deionized with a Milli-Q system manufactured by Millipore (hence mQH_2_O).

### Preparation of stock solutions and protein standards

A 50 mM ammonium bicarbonate solution was prepared by weighing NH_4_HCO_3_ and dissolving it in mQH_2_O. Similarly, Tris buffer pH 10 was prepared by mixing TRIZMA base and calcium chloride dehydrate in mQH_2_O. To obtain a 100 mM Tris-DTT solution, DTT was dissolved in Tris buffer pH 10, just before use. A 200 mM IAA solution was prepared by weighing and resuspending IAA in mQH_2_O. The supplied lyophilized Rapigest powder was reconstituted in 50 mM ammonium bicarbonate to obtain a Rapigest 0.1% solution. A solution of BSA was prepared by diluting the stock solution of 2 mg/ml to 4000 ng/ml in mQH_2_O.

Stock solutions of the PEPotec peptides were prepared at 100 pmol/µl. Different mixes of stable isotope-labeled peptides were prepared at 20 ng/ml of each peptide for the LC–MS/MS setting optimization. The supplied lyophilized trypsin was thawed and reconstituted in the supplied resuspension buffer to obtain a 100 ng/µl solution. Just before use, the trypsin solution was activated by incubating it for 10 min at 37 °C. The buffers used for the MRM assay were prepared as follows: 0.1% FA in mQH_2_O and 0.1%FA in Acetonitrile (ACN) by adding FA in respectively (resp.) mQH_2_O and ACN.

As no urine blank control samples were commercially available, a non-related urine test sample was used, based on its low basal intensities measured of the different investigated proteins. This test sample was spiked with different concentrations of commercially available proteins to create standard curves in order to test the linearity for each protein. The concentrations of the standard curves were selected on the expected ranges of concentrations of the proteins naturally occurring in urine and on the values obtained in previous studies with the same children^28,33,50–57^. In addition to linearity, reproducibility, LOD, LOQ and carry-over were investigated with this urine test sample. Stability of the peptides was assessed by injecting the same control sample, spiked with a mix of peptides and stored at − 20 °C, before each run. However, not all investigated proteins (i.e. not OPN) could be spiked at the required high concentrations, mimicking the naturally abundant amounts present in urine samples, due to the high purchasing costs and the limited budget of this feasibility study. For this protein, the validation was not completely executed.

### Tryptic digestion

Urine samples were thawed on ice overnight. Cells and tissue debris were removed by low speed centrifugation (5000 rpm). 4.5 ml of methanol was added to 500 µl urine and stored at − 20 °C to allow protein precipitation to occur overnight. After centrifugation, the supernatant was removed and the pellet air dried. The pellet was resuspended in 200 µl Tris-DTT and 30 µl Rapigest 0.1% was added. A known amount of the synthetic stable isotope-labeled AQUA peptides (corresponding with 50 ng/ml of each peptide) was spiked into the samples. After thorough mixing, dissolving, heating (for 10 min in an oven at 100 °C) and cooling down, 30 µl of IAA was added. The pH was checked during additional cooling down, and was expected to be between 7 and 9. Finally Trypsin (30 µg, 100 ng/µl) was added and the samples were digested overnight at 37 °C. After digestion, FA was added to quench the trypsin activity by incubation at room temperature for 30 min. The sample was then transferred to a high recovery vial for the MRM assay. Non-human bovine serum albumin (BSA, 66 kDa) was included as a control for digestion efficiency. The same BSA concentration was spiked in each sample undergoing trypsin digest. If the digest went through, the BSA was successfully cleaved, leading to a constant signal, representing the abundance of the cleaved BSA peptides. Unsuccessful trypsin digest would lead to a much lower or absent signal. To correct for this potential variation of this biological process, the obtained BSA signals were used to normalize (by simple division) the signal of the other peptides.

### UPLC–MS/MS method development and validation

The proteomics analyses were carried out using a TQ-S triple quadrupole instrument coupled to an Acquity UPLC instrument both from Waters. Samples (10 µl) were injected on a peptide BEH C18 column (1.0 mm × 100 mm, 1.7 µm, 130 Å). The peptides were eluted at 0.2 ml/min using a gradient of solvent A (water with 0.1% FA) and solvent B (ACN with 0.1% FA) increasing from 2% at injection to 60% B at 11 min with subsequent cleaning and regeneration of the column in the following 4 min. The peptide analysis was done in positive MRM mode with capillary voltage at 3.0 kV, cone voltage at 30 V, source offset at 60 V, source temperature at 150 °C, desolvation temperature at 550 °C, cone gas flow at 150 l/h, and desolvation gas flow at 1000 l/h.

The relative quantification was obtained using the TargetLynx data analysis software (Masslynx 4.1 SCN950, Waters, www.waters.com). To correct for the trypsin digest efficiency, the response of each peptide was adjusted with the response obtained for the BSA peptides present in the same urine sample.

The limit of detection (LOD) and quantification (LOQ) were determined, based on the signal-to-noise (S/N) approach, respectively S/N = 3 and S/N = 10. The S/N ratio was determined by comparing measured peak intensity from a sample with a known concentration of spiked protein with the intensity of the noise flanking the peak. The spiked synthetic stable isotope-labeled peptides have a known mass difference from the native proteotypic peptides, allowing the relative endogenous concentration measurement by comparing their signal with this of the exogenous labeled species.

In a next step, the linearity was investigated for CC16, RBP4, HSA, β2M, NF-κB, MYO and OPN. Mixes of the stable isotope-labelled peptides were spiked at 50 ng/ml in urine together with a range of concentrations of the intact proteins to create a standard curve. The selected concentration range of each protein was depending on their expected naturally occurring concentrations in urine. The reproducibility was investigated for CC16, RBP4, HSA, NF-κB, MYO and β2M by measuring three blank urine samples spiked with three specific concentrations, ranging from low to high, of the protein for three consecutive times and on three different days^58^. The criteria for acceptance was set to a coefficient of variance of less than 25% for the lowest concentration and less than 20% for the other concentrations. This is slightly higher than the nonbinding recommendations of the FDA for bioanalytical method validation based on a chromatographic run (respectively 20% and 15%)^59^. This is explained by the fact that our method consists of a chromatographic run and a prior biological process, i.e. the trypsin digestion, which can add some additional variation. Also the possible carry-over in the chromatography system was checked. Once the method was validated, all the urine samples of the children underwent trypsin digest (as described in the paragraph above). Each sample was spiked with a fixed concentration (50 ng/ml) of a mixture of stable isotope-labelled peptides that were selected. Protein abundances were measured with the validated MRM method and compared with the results obtained with the immunoassays which were available for CC16, β2M, HSA, RBP4. Also correlations between the different proteins and with creatinine were studied.

### Data analysis

All the raw data acquired from the triple quadrupole MS were imported into Targetlynx software for further quantification. Manual inspection of all the data was performed to ensure accurate peak integration and correct peak detection. For all peptides with multiple transitions, the peak area ratio of the transitions was checked for the different proteins. Similar peak area ratio ranges were observed during the validation of the method and the measurements of the children’s urine samples of the field study. The results were then exported to Microsoft excel for further analysis, such as linear regression, reproducibility, and plotting. The association between the different variables was evaluated by Pearson’s correlation analysis using JMP software (Pro 14.3.0, SAS, www.sas.com). Data of reproducibility are presented as mean coefficients of variance ± standard deviations for three independent experiments.

### Ethics approval and consent to participate

The Ethics Committee of the Faculty of Medicine of the Catholic University of Louvain approved the study protocol, which complied with all applicable requirements of international regulations (Registration number B403201734310).

## Results

### UPLC–MS/MS method development

The first step of the workflow for the MRM experimental design was the selection of the appropriate proteotypic peptides of the targeted proteins^60^. The amino sequences of CC16 (accession number P11684), β2M (accession number P61769), OPN (accession number P10451), RBP4 (accession number P02753), HSA (accession number 02768), MYO (accession number P02144), NF-κB (accession number Q04206) and BSA (accession number P02769) were found on www.uniprot.be. In silico cutting of the amino sequence of the proteins with trypsin was performed with Peptide Cutter^61^, resulting in a list of possible peptide sequences. The selection of the most appropriate surrogate peptide for a given protein is not trivial, since two major conditions need to be met: uniqueness of the peptide sequence and a good detectability in LC–MS/MS, the latter being influenced by its physicochemical properties. The selected peptide needs to be relatively small (6 to 20 amino acids long), should preferentially not contain amino acids with high tendency for chemical induced modification (oxidation or deamidation, like methionine or asparagine) and internal tryptic sites (lysine or arginine) should be avoided. Furthermore, to observe consistent signals, the selected candidates should have good LC–MS/MS properties, including proper chromatographic behavior (avoiding highly hydrophobic or hydrophilic peptides) and a high ionization efficiency^62^. In addition, BLAST^63^ was used to confirm the uniqueness of a specific peptide^64^. For each protein, preferably two candidate proteotypic peptides needed to be selected based on the criteria mentioned above. However, it was not always possible to meet each criterion. In case of a small protein like CC16, the in silico selection yielded initially only three potential proteotypic peptide candidates. After thorough testing and optimization, explained in the next paragraph, only one proteotypic peptide (with two transitions) remained suitable for relative quantification of CC16. This peptide was found not to be completely unique and also occurs in *Bifidobacterium longum NCC2705* and, less pertinent, in the Japanese maqaque *Macaca fuscata fuscata*. Nevertheless, the possible impact of this non-uniqueness was further investigated in downstream analysis and was found not to be problematic in the case of this study. An overview of all the initially selected proteotypic peptides for the MRM method as well as the selection procedure can be found in the Supplementary table S1 online.

In a first stage, these labelled synthetic peptides were infused into the triple quadrupole instrument to optimize a number of MS parameters (precursor and daughter mass-to-charge (*m*/*z*) ratios, collision energy (CE) for fragmentation and cone voltage). Default settings (mother and daughter *m*/*z* ratios, CE, cone voltage, etc.) of the UPLC–MS/MS method were in a first step optimized using the IntelliStart software (Masslynx 4.1 SCN950, waters, www.waters.com). This software automatically determines the most appropriate set of these parameters when infusing the peptides directly into the mass spectrometer. These values were further fine-tuned by comparing the obtained peak intensities after stepwise variation of the parameters when injecting samples of peptide mixes over the column. The varying intensities obtained for the optimization of the collision energy are illustrated for two proteins (CC16 and RBP4) in the Supplementary Fig. S2 online. The CE value corresponding with the highest intensity for a peptide was further used in the final UPLC–MS/MS method.

Subsequently, the peptides were injected into the UPLC-instrument to optimize the chromatographic gradient for optimal separation of the targeted peptides and determine their respective retention time (see Table 1). In a next step the trypsin digest, resulting in the fragmentation of the proteins into peptides, was optimized (e.g. concentration trypsin, IAA, DTT,…) using commercially available intact proteins spiked in different volumes of a test urine sample. The use of 500 µl of urine resulted in higher obtained peptide signals, which is illustrated in the Supplementary Fig. S3 online. The peptides yielding the highest intensities after digestion were selected for further method validation (see Table 1). For each protein and per peptide, preferably two transitions, characterized by their parent and daughter ion mass-to-charge ratios, were selected. However, it was not always possible to obtain satisfying high intensity signals for two transitions per proteotypic peptide. In that case, a transition selected from the second proteotypic peptide was used. Finally, results were analyzed for at least two transitions (from the same or from a different labelled proteotypic peptide). One was used for relative quantification while the other was used to confirm the result.

**Table 1:** Selection of proteotypic peptides used for relative quantification by MRM analysis.

Protein	Proteotypic peptide	Transition (*m*/*z*)				Fragment ion Q3	Collision E	Cone V	Retention time (min)
		Native peptide		Labelled peptide					
CC16	EAGAQLK	358.9	516.6^a^	362.9	524.6	Y-ion	10	30	4.5
		358.9	260.5^b^	362.9	268.5	Y-ion	7	30	4.5
RBP4	YWGVASFLQK	599.6	849.7^a^	603.6	857.7	Y-ion	9	30	8.4
	LIVHNGYCDGR	435.2	539.6^b^	438.5	544.6	Y-ion	9	30	5.5
β2M	VNHVTLSQPK	375.0	244.3^a^	377.7	252.3	Y-ion	20	30	5.3
		375.0	459.0^b^	377.7	467.3	Y-ion	12	30	5.3
OPN	GDSVVYGLR	483.3	607.2^a^	488.3	617.2	Y-ion	15	30	6.4
		483.3	508.2^b^	488.3	518.2	Y-ion	15	30	6.4
HSA	SLHTLFGDK	340.0	319.3^a^	342.7	327.3	Y-ion	12	30	6.7
		340.0	466.3^b^	342.7	474.3	Y-ion	12	30	6.7
	HPYFYAPELLFFAK	581.9	483.1^b^	584.6	487.1	Y-ion	15	30	9.0
MYO	HGATVLTALGGILK	451.0	367.6^a^	453.7	367.6	B-ion	10	30	8.2
		451.0	487.6^b^	453.7	495.6	Y-ion	16	30	8.2
NF-κB	LPPVLSHPIFDNR	502.3	648.1^a^	505.6	653.1	Y-ion	12	30	7.4
		502.3	289.4^b^	505.6	299.4	Y-ion	20	30	7.4
BSA	LGEYGFQNALIVR	740.9	813.8^a^	745.9	823.8	Y-ion	30	30	7.9
		740.9	1018.2^b^	745.9	1028.2	Y-ion	30	30	7.9

^a^Selected for method validation and relative quantification

^b^Selected for confirmation of result; *m*/*z*: mass-to-charge ratio; *E* energy; *V* voltage.

### UPLC–MS/MS method validation

Table 2 summarizes the linearity, the reproducibility, LOD and LOQ obtained with the MRM method for the different proteins. The standard curves describing the linearity and the peaks used for signal-to-noise and subsequent LOD and LOQ determination can be found in the Supplementary Fig. S4 and S5 online. The linearity was assessed for all proteins by supplementing a urine test sample with each of the commercially available recombinant proteins to a final concentration range similar to what is naturally occurring in urine. Comparison of the expected to the observed concentrations confirmed the linearity across the analytical measurement range with an r^2^ of > 0.98 (see Supplementary Fig. S4 online). The reproducibility of the three independent MRM analyses was done on three different days by using a test urine sample spiked which three different concentrations of pooled proteins. The reproducibility testing yielded CV values below 20% for almost all tested concentrations of urinary CC16 (U-CC16), urinary RBP4 (U-RBP4), urinary HSA (U-HSA), urinary myoglobin (U-MYO), urinary NF-κB (U-NF-κB) and urinary β2M (U-β2M) for the inter-sample (between a sample prepared in threefold), intra-sample (between threefold measurement of the same sample) and inter-day variability. Only the inter-sample and inter-day variation for U-β2M at 50 ng/ml was slightly higher (23%). For U-OPN, the reproducibility could not be investigated, due to the high costs needed for preparing multiple samples spiked with the required amounts of the OPN protein to mimic its abundant presence in urine. The LOQ ranged from 0.3 up to 42.8 ng/ml for the different proteins investigated. No carry-over was observed and the stability of the proteotypic peptides was retained throughout the different injections (data not shown). Typical MRM chromatograms, representing the labelled and native peptides co-eluting in the analyzed urine samples are illustrated in Supplementary Fig. S6 online. The raw data of the validation and of the children’s urine samples were uploaded in a public repository (PeptideAtlas with the dataset identifier PASS01660). The transition list, listing all the used parameters of the MRM method can be found in the Supplementary Table S7 online.

**Table 2:** Overview of the linearity, reproducibility, LOD and LOQ obtained for each protein.

Protein	Linearity		Reproducibility (%)			LOQ (ng/ml)	LOD (ng/ml)
	r^2^	Conc. (ng/ml)	Intra-sample	Inter-sample	Inter-day		
U-CC16	0.999	4	14.8 ± 5.8	16.4 ± 3.2	16.6	0.5	0.2
		25	5.6 ± 2.5	8.2 ± 0.9	5.0		
		100	1.8 ± 0.9	2.9 ± 0.6	3.1		
U-RBP4	0.999	20	10.0 ± 5.2	14.4 ± 0.3	15.1	0.8	0.3
		100	8.8 ± 5.5	10.8 ± 3.6	12.0		
		500	7.5 ± 2.8	8.4 ± 0.5	8.1		
U-β2M	0.996	50	10.3 ± 9.1	23.2 ± 3.1	22.8	13.3	4.4
		150	3.6 ± 6.2	10.3 ± 0.7	12.8		
		750	5.8 ± 5.3	13.5 ± 2.8	13.7		
U-HSA	0.998	1000	8.6 ± 6.3	18.0 ± 0.9	17.7	42.8	14.3
		5000	4.9 ± 1.2	4.0 ± 0.1	4.3		
		25,000	6.8 ± 2.8	13.0 ± 0.2	12.2		
U-MYO	0.999	4	11.7 ± 6.2	12.9 ± 0.0	13.4	1.5	0.5
		25	4.5 ± 1.7	8.8 ± 0.4	8.5		
		100	2.8 ± 2.0	3.6 ± 1.1	3.9		
U-NF-κB	0.999	4	11.3 ± 3.2	13.9 ± 3.0	15.9	0.3	0.1
		25	6.3 ± 2.6	12.5 ± 0.2	12.1		
		100	2.8 ± 1.6	8.8 ± 1.0	8.5		
U-OPN	0.989	NA^a^	NA^a^	NA^a^	NA^a^	5.4	1.8

^a^Due to the high costs needed for obtaining the required amounts of the OPN protein to mimic its abundant presence in urine (U-OPN), it was not possible to do a complete method validation. *Conc*. Concentration, *NA* not available, *LOD* limit of detection, *LOQ* limit of quantification.

The linearity is determined by r^2^ of the curve for the specified concentration range (ng/ml). The reproducibility is characterized by a coefficient of variance (%) ± standard deviation. The LOD and LOQ are derived from the signal-to-noise ratio and are expressed in ng/ml.

### Comparison of the values obtained between MRM and LIA

The MRM method was cross-validated by measuring a total of 72 urine samples from a group of children and by comparing them with the results previously obtained from immunoassays (LIA) of four proteins (CC16, β2M, RBP4 and HSA). Data for MRM quantification in urine were missing for 8 subjects for CC16, 4 subjects for β2M, 6 subjects for RBP4, 6 subjects for HSA, 4 subjects for OPN, due to very low concentrations below LOQ or a technical issue during the digest protocol. The latter was true for 4 samples based on the observed BSA signal that was used as a quality control parameter for the digestion process. Although the MRM method allows a very low LOQ, quantification data for NF-κB and MYO were not obtained, due to their low abundance/absence in these urine samples from children.

**Figure 1:**
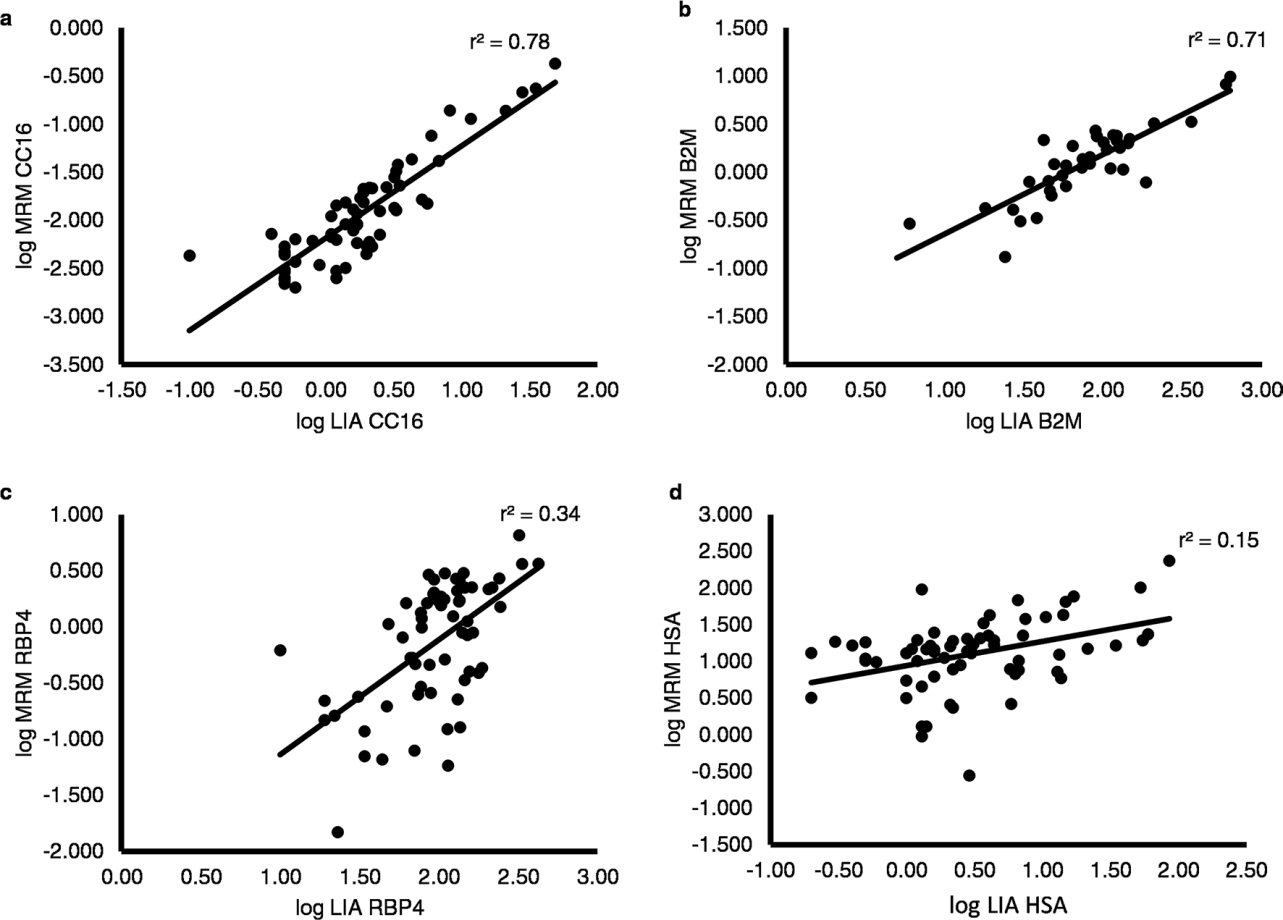
Comparison of the protein values obtained between MRM and immunoassay: Correlation (r^2^) between log-transformed CC16 (**a**), β2M (**b**), RBP4 (**c**) and HSA (**d**) quantification measured by Latex Immunoassay (LIA) versus MRM.

Our MRM method correlated to the LIA with a good correlation of r^2^ = 0.78, r^2^ = 0.71 for resp. CC16 and β2M and a rather poor correlations of r^2^ = 0.34 and r^2^ = 0.15 for RBP4 and HSA (log–log comparison) (Fig. 1). The good correlation between the MRM and the LIA data of CC16 implies that the non-uniqueness of the used peptide, as mentioned above, does not interfere in the selective and accurate determination of the U-CC16 levels with the MRM method.

### Correlations between measured proteins and correlation with creatinine in view of finding an adjuster for renal handling and diuresis

Finally, the univariate associations between the different biomarker candidates detected in urine and their potential adjusters was investigated. Table 3 gives an overview of the Pearson correlations between the log-transformed levels of the different proteins. As expected, between the biochemically similar proteins U-RBP4 and U-β2M, a high agreement was seen with a r of 0.88 (*p* < 0.001). The adjustment by simple division with the potential adjuster (Table 3(A)) was compared with the adjustment on the basis of the regression coefficient between the two analytes (Table 3(B)). The values of U-CC16 correlated with the potential adjusters, β2M and RBP4 (resp. r = 0.58, *p* < 0.001 and r = 0.48, *p* < 0.001), but according to the method of adjustment, i.e. simple division or adjusting on the basis of the regression coefficient, the correlation pattern differed noticeably. For instance, when expressing U-CC16 as a ratio to U-β2M, not surprisingly, there was a good correlation between U-CC16/U-β2M and unadjusted CC16 (r = 0.69, *p* < 0.001). However, some residual correlations persisted with the adjuster U-β2M (r = −0.18, *p* = 0.15), but also with U-HSA (r = −0.25*, p* < 0.05) and U-RBP4 (r = −0.20, *p* = 0*.*11). By contrast, when adjusting U-CC16 using the observed regression coefficient between U-βM2 and U-CC16, no residual correlation with U-β2M (r = 0.00, *p* = 0*.*99) and lower residual correlations for U-HSA (r = −0.14, *p* = 0*.*28) and U-RBP4 (r = −0.04, *p* = 0*.*75) were observed. Of note, the adjustment of U-CC16 as a ratio to β2M or using the regression coefficient with β2M almost abolished the correlations with U-Creat (r = 0.02, *p* = 0*.*89 and 0.07, *p* = 0*.*58, resp.). Additionally, an even better correlation between U-CC16 adjusted with U-β2M and U-CC16 was obtained (r = 0.81, *p* < 0*.*001). Similar patterns of correlations were observed for U-RBP4 as adjuster but with a tendency to more residual correlations, in particular with U-β2M and U-Creat (Table 3). OPN, a protein with similar size as CC16, also showed similar correlation results as obtained for U-CC16, although less pronounced (Table 3).

**Table 3:** Pearson’s correlation coefficient between the detected urinary proteins biomarkers.

	CC16	RBP4	β2M	OPN	Creat	HSA
**(A)**						
CC16	1.00*					
RBP4	0.48*	1.00*				
β2M	0.58*	0.88*	1.00*			
OPN	0.25^$^	0.49*	0.52*	1.00*		
Creat	0.23	0.27^$^	0.30^$^	0.14	1.00*	
HSA	0.27^$^	0.66*	0.74*	0.33^$^	0.25^$^	1.00*
CC16-RBP4	0.53*	− 0.48*	− 0.28^$^	− 0.23	0.06	− 0.40^#^
CC16-β2M	0.69*	− 0.20	− 0.18	− 0.09	0.02	− 0.25^$^
CC16-creat	0.73*	0.31^$^	0.32^$^	0.21	− 0.50*	0.07
CC16-HSA	0.64*	− 0.14	− 0.03	0.025	0.03	− 0.53*
OPN-RBP4	− 0.27^$^	− 0.32^#^	− 0.47*	0.67*	− 0.09	− 0.39*
OPN-β2M	− 0.25	− 0.30^$^	− 0.22	0.72*	− 0.28^$^	− 0.18
OPN-creat	0.04	− 0.31^$^	0.072	0.84*	− 0.43*	0.00
OPN-HSA	− 0.17	− 0.50*	− 0.44*	0.07	− 0.15	− 0.87*
RBP4-creat	0.25^$^	0.73*	0.56*	0.37^#^	− 0.47*	0.43*
HSA-creat	0.07	0.38^#^	0.35^#^	0.04	− 0.53*	0.68*
**(B)**						
CC16	1.00*					
RBP4	0.48*	1.00*				
β2M	0.58*	0.88*	1.00*			
OPN	0.25^$^	0.49*	0.52*	1.00*		
Creat	0.23	0.27^$^	0.30^$^	0.14	1.00*	
HSA	0.27^$^	0.66*	0.74*	0.33^#^	0.25^$^	1.00*
CC16-RBP4	0.88*	0.00	0.17	0.01	0.17	− 0.14
CC16-β2M	0.81*	− 0.04	0.00	− 0.01	0.07	− 0.14
CC16-creat	0.99*	0.47*	0.55*	0.25^$^	0.09	0.24
CC16-HSA	0.96*	0.30^$^	0.41*	0.19	0.17	0.00
OPN-RBP4	− 0.10	0.00	− 0.22	0.87*	0.00	− 0.19
OPN-β2M	− 0.12	− 0.14	0.00	0.85*	− 0.23	− 0.01
OPN-creat	0.24	0.48	0.31^$^	1.00*	0.09	0.14
OPN-HSA	0.11	0.27^$^	0.23	0.94*	0.05	0.00
RBP4-creat	0.45*	0.98*	0.86*	0.49*	0.11	0.64*
HSA-creat	0.23	0.61*	0.62*	0.14	0.10	0.99*

Pearson’s correlation coefficient between the detected urinary proteins biomarkers (CC16, OPN) in urine and their potential adjusters (β2M, RBP4, HSA, creatinine) when analytes were (A) adjusted by division with the potential adjusters or (B) adjustment based on the regression coefficient of the two analytes.

^$^< 0.05; ^#^< 0.01; *< 0.001. All parameters were log transformed. Statistical significance was evaluated by two-tailed t-test.

Usually urinary proteins are expressed per gram of creatinine (by division). Here, U-CC16, expressed as a ratio to U-Creat, correlated very well with the unadjusted U-CC16 (r = 0.73, *p* < 0*.*001). However, again this type of adjustment did not completely abolish the association of the urinary biomarker with U-Creat, but rather switched to a negative correlation (r = −0.50, *p* < 0*.*001). Adjusted on the basis of the regression coefficient with U-Creat, U-CC16 correlated highly with the unadjusted U-CC16 (r = 0.99, *p* < 0*.*001), but this leads to very significant residual correlations with U-RBP4 (r = 0.47, *p* < 0*.*001), U-β2M (r = 0.55*, p* < 0*.*001) and U-HSA (r = 0.24, *p* < 0*.*05). Such significant residual influences of the diuresis (from U-Creat), glomerular filtration (from U-HSA) or tubular reabsorption (from U-β2M or U-RBP4) after adjusting with U-Creat are not observed when adjusting with U-RBP4 and even less with U-β2M. Adjustment on the basis of U-β2M or U-RBP4, abolishes the influence of both diuresis and tubular reabsorption, with a small influence of glomerular filtration remaining.

## Discussion

To dispose of a method to simultaneously quantify different biomarkers in a noninvasive biofluid would significantly facilitate the biomonitoring of specific target groups at risks. In the context of monitoring of children, this biofluid sample is preferably a noninvasive one, such as urine. Our developed MRM method resulted in the ability to selectively measure multiple distinct proteins in urine by targeting sequence-specific tandem MS fragmentations of proteotypic peptides. These proteins consisted of several protein biomarkers related to respiratory health that were selected from literature, i.e. CC16, OPN and NF-κB, as well as of a number of potential urinary adjusters, i.e. β2M, RBP4 and MYO and a marker of renal dysfunction (HSA). The MRM method was completely validated for all proteins and partially for OPN. It allowed the simultaneous relative quantitation for all the proteins in the low and high ng/ml concentration ranges, with LOQ’s ranging from 0.3 to 42.8 ng/ml. The investigation of the linearity and reproducibility yielded good results for all tested proteins, except for U-OPN which was not included in the complete validation, due to cost reasons. However, the presence or absence of OPN as well as differences between high and low concentrated samples could definitely be detected. Nevertheless, all other proteins yielded a very good linearity (r^2^ > 0.99) and standard deviations < 25% for the lowest concentrations and < 15% for the middle and highest concentrations for inter-day reproducibility. These values are very satisfactory, considering that a biological process like the trypsin digestion is part of the urine sample preparation procedure.

In the context of this study of investigating potential urinary respiratory health biomarkers, the MRM method was further validated by applying our method to urine samples of children, ranging from 9 to 11 years. Comparison of the MRM results was done with available classical immunochemistry assay (LIA) data for β2M, CC16, RBP4 and HSA. The MRM quantification demonstrated a reasonably good relative quantitative performance for U-CC16 and U-β2M, which matched (r^2^ ≥ 0.70) with the respective immunoassays results, confirming the validity and robustness of this MRM-method. The correlations between MRM and the immunoassays for RBP4 and HSA for the selected transitions were lower with r^2^ = 0.34 and r^2^ = 0.15, respectively. However, HSA contains three domains, each potentially present in relatively different abundances in urine due to fragmentation or modification of the intact albumin^65^. This can have a significant impact on the performance of the affinity-based assays^66,67^. In addition, performance is highly dependent on the location of the antibody epitope. While the immunoassay, based on the use of a polyclonal antibody, does not make the distinction between each domain, our MRM method used one proteotypic peptide, targeting one domain of HSA. In addition, it has already been shown previously that there could be discordance among various methods used for the determination of the level of U-HSA in clinical and intervention studies^68^. Similarly, the RBP4 protein is known to exist in different isoforms in urine and therefore presents unique challenges. Truncation at the C-terminus and post-translational cleavages or modifications can significantly influence its detection^31,69^. These biological processes can explain the lower correlation between both techniques for RBP4.

Myoglobin (MYO), which we initially also selected as a potential adjustment candidate for dilution and renal handling, was despite its low LOD of 0.5 ng/ml not detected in the measured samples. This could be anticipated, as myoglobin is present in very low concentrations (or even absent) in the urine of healthy people^33^. However, in the case of other studies, for instance investigating cardiac or skeletal injury, where increased levels of myoglobin are expected in the urine^70,71^, this MRM method could be applied.

The MRM method was also developed for NF-κB and partially for OPN. Several studies identified NF-κB as a good potential biomarker for asthma severity and lung inflammation in several tissues and fluids such as NALF^24–26^ but was not yet described in urine. Our MRM method was not able to detect it in the urinary samples of the tested children, despite the very low detection limit of the method, of 0.1 ng/ml. This might be due to the very low levels of NF-κB present in urine of healthy persons. Moreover, no other studies involving respiratory health yet reported the successful detection of NF-κB in urinary samples. This suggests that urine might not be the adequate noninvasive biofluid to investigate NF-κB as respiratory health biomarker. However, in other noninvasive biofluids, such as NALF, the MRM method could potentially be applied for the detection of NF-κB, as elevated NALF-NF-κB levels were found to be associated with asthma^27^. The detection of OPN in urine, as a noninvasive alternative, in the respiratory health context, has also not yet been described in other studies. Additional research is needed to demonstrate the relationships between the levels found in urine and the physiological and pathophysiological states. Now that the MRM method has been developed, it will be possible to conduct this type of studies in a large scale setting. Alternatively, the method could also be used for the detection of NF-κB and OPN in studies involving other health conditions, such as chronic glomerulonephritis or nephriolitiasis where resp. U-NF-κB and U-OPN levels were successfully detected^30,72^.

The main advantage of this MRM method is the simultaneous measurement of different proteins. This is of interest because multiple urinary protein biomarkers can be measured at once, in addition to the simultaneous measurement of potential urinary adjusters. Confounding factors such as variations in tubular reabsorption, glomerular filtration and diuresis can therefore be taken into account. The results from this study confirm that the abundances of U-CC16 or U-OPN are considerably altered by the method used for urine concentration adjustment^28,73,74^. By dividing by creatinine, the effect of diuresis is not completely abolished and even reverses the direction of the association. This adjustment, however, is used systematically in most studies in literature, and questions to what degree the reported associations are accurate. The adjustment with creatinine, based on the regression coefficient improves the results and was previously successfully applied in Wang et al.^28^. However, in our study, using creatinine still leaves some residual associations with U-β2M, U-RBP4 and/or U-HSA. This means that the influences of tubular reabsorption or glomerular filtration are not completely abolished neither. This is in contrast with using an adjustment based on the regression coefficient of U-RBP4 and especially U-β2M for determination of the actual urine levels of CC16, OPN or potentially any other LMW molecules. Almost no residual associations linked with tubular reabsorption, glomerular filtration or diuresis persisted. This adjustment seems to compensate for the different potential confounding effects that could occur with LMW urinary proteins in the kidney and leads to our suggestion for systematic adjustment of U-CC16, U-OPN or other LMW urinary proteins with U-βM2 or U-RBP4 based on their regression coefficient. Adjustment of U-CC16 with U-RBP4 has been applied in previous studies^75,76^. Based on this adjustment, these previous small scale studies, using immunoassays for urinary protein quantification, showed significant correlations between U-CC16 and the CC16 A38G genotype^76^ or in subjects with bronchiolitis^75^. However, β2M has been more challenging to determine up till now. β2M is a small and cationic protein which degrades quickly in acid urine environment, potentially hampering accurate quantification with immunoassay. This bottleneck is avoided when using MRM, as this mass spectrometry method is based on the quantification of signature peptides of the investigated protein. Our MRM method gives the advantage to detect β2M, even degraded and fragmented, and provides an accurate and simultaneous estimation of U-β2M, U-RBP4 and U-CC16 in multiple urine samples. Additionally, this method allowed the identification of β2M and RBP4 as suitable adjusters for renal handling and diuresis when measuring LMW proteins.

The development of this tool, i.e. a high throughput MRM method offering simultaneous measurement of urinary proteins and its potential adjusters, allows the set-up of future studies with larger subject groups, to further investigate if urine can be used for measuring respiratory health biomarkers (potentially U-CC16, U-NF-κB or U-OPN) in children. This type of studies could be stratified according to disease or genotype impacting the respiratory health, while using the appropriate adjustment to demonstrate relevant associations. Alternatively, the method can be used for biomonitoring other aspects of human health. Very recently, in relation to the COVID-19 pandemic, HSA and MYO have already been cited as proteins of major interest, either to estimate the mortality risk (HSA)^77^ or for monitoring patients undergoing treatments like hemodialysis or who are diagnosed with the complication rhabdomyolysis (MYO)^33,78^. Additionally, taking into account the central role of the lungs as a primary target in COVID-19, it is not unlikely that other proteins like e.g. CC16 can also become of major importance to monitor this disease.

One of the limitations of our MRM method could be that the abundance determination is relative and not absolute. However, relative quantification gives sufficient insight when comparing protein levels in samples from different conditions (healthy versus diseased samples, exposed versus non exposed). A further extended validation of the MRM method would allow the absolute quantification of potential biomarkers and could however be more advantageous in some cases, e.g. for diagnostic purposes in hospitals. Moreover, if necessary in future studies, the method could further be complemented with more newly discovered protein candidates, potentially playing a role in respiratory health or in other health conditions.

## Conclusions

We developed and validated a MRM-method able to analyze urine samples in the low and high ng/ml concentration ranges. The MRM method was used to allow the simultaneous relative quantification through MRM analysis of a selection of 7 different proteins, initially selected as potential markers of respiratory health or potential adjustment candidates for diuresis and renal handling of LMW proteins such as CC16 and OPN. However, in the context of respiratory health investigation, they were not all detected in urine, due to their low naturally occurring presence (or even absence). Nevertheless, the MRM method could be applied for investigating if urine can be used as a good surrogate to serum and to analyze urine samples in the context of other health conditions, characterized by altered levels of these proteins. The biggest advantage of this MRM-method compared to the immunoassay is that the 7 proteins are measured simultaneously, contrarily to the numerous simplex immunoassays that would be required and needed to be available. This multiplex MRM method is not limited by these inter-assay variations, therefore reflecting more accurately the true abundances within the samples, including the adequate adjustment.

When applying this method on a cohort of urine samples of children, good correlations with the immunoassay results were found for CC16 and β2M. Using β2M as adjuster compensates for the confounding effects linked to the renal handling and diuresis and could therefore be proposed as systematic adjuster when investigating LMW proteins in urine. Measuring biomarkers and proteins for adjustment in noninvasive biofluids, using inexpensive multiplex high-throughput methods based on MRM will contribute to understanding the complex relationship between cause and effect in future studies. This is also of critical importance for health care management purposes, public health decision making, and primary prevention activities.

## Supplementary information

Supplementary Informations.

## Data Availability

The data generated during and/or analyzed during the current study are available in the PASSEL repository (PeptideAtlas), with the dataset identifier PASS01660, http://www.peptideatlas.org/PASS/PASS01660.

## Abbreviations

ACN: Acetonitrile
β2M: Beta-2-microglobulin
BSA: Bovine serum albumin
CC16: Club cell protein
CE: Collision energy
DTT: dl-Dithiothreitol
FA: Formic acid
HSA: Human serum albumin
IAA: Iodoacetamide
LC–MS/MS: Liquid chromatography coupled with tandem mass spectrometry
LIA: Latex immunoassay
LMW: Low-molecular-weight
LOD: Limit of detection
LOQ: Limit of quantification
*m*/*z*: Mass-to-charge ratio
MRM: Multiple reaction monitoring
MS: Mass spectrometry
MYO: Myoglobin
NALF: Nasal lavage fluid
NF-κB: Nuclear factor-kappa B
NH_4_HCO_3_: Ammonium bicarbonate
OPN: Osteopontin
RBP4: Retinol binding protein 4
resp.: Respectively
S/N: Signal-to-noise
TFA: Trifluoroacetic acid
U-β2M: Urinary β2M
U-CC16: Urinary CC16
U-HSA: Urinary HSA
Y-MYO: Urinary myoglobin
U-NF-κB: Urinary NF-κB
U-OPN: Urinary OPN
U-RBP4: Urinary RBP4
UPLC: Ultra performance liquid chromatography
