# Supplementary material for: Development of a multiplex mass spectrometry method for simultaneous quantification of urinary proteins related to respiratory health
Source: Sci Rep. 2021 May 12;11:10107. doi: 10.1038/s41598-021-89068-9 (PMC8115669; doi:10.1038/s41598-021-89068-9)
Supplement: Supplementary file 1 — Supplementary Informations. [file 41598_2021_89068_MOESM1_ESM.pdf]

Development of a multiplex mass spectrometry method for simultaneous quantification of urinary proteins related to respiratory health

Sarah J. D. Nauwelaerts, Nancy H. C. Roosens, Alfred Bernard, Sigrid C. J. De Keersmaecker, Koen De Cremer

Table S1: overview of proteotypic peptides and selection procedure (infusion, trypsin digest, selection of best transitions)

protein		infusion	high trypsin digest efficiency	most intense transitions selected (1-2)
CC16	LVDTLPQ[K]	passed	failed	NA
	VIETLLMDTPSSYEAAMELFSPDQDM[R]	failed	NA	NA
	EAGAQL[K]	passed	passed	1st; 2nd
RBP4	FSGTWYAMA[K]	failed	NA	NA
	DPEGLFLQDNIVAEFSVDETGQMSATA[K]	failed	NA	NA
	YWGVASFLQ[K]	passed	passed	1st
	DPNGLPPEAQ[K]	passed	failed	NA
	LIVHNGYCDG[R]	passed	passed	2nd
MYO	VEADIPGHGQEVLI[R]	passed	passed	NA
	GHPETLE[K]	passed	passed	NA
	ASEDL[K]	passed	failed	NA
	HGATVLTALGGIL[K]	passed	passed	1st; 2nd
	HPGDFGADAQGAMN[K]	passed	passed	NA
NF-κB	SAGSIPGE[R]	passed	passed	NA
	INGYTGPGTV[R]	passed	passed	NA
	PHPHELVG[K]	passed	failed	NA
	LPPVLSHPIFDN[R]	passed	passed	1st; 2nd
	TPPYADPSLQAPV[R]	passed	passed	NA
HSA	HPYFYAPELLFFA[K]	passed	passed	2nd
	SLHTLFGD[K]	passed	passed	1st
	VPQVSTPTLVEVS[R]	passed	passed	NA
B2M	VNHVTLSQP[K]	passed	passed	1st; 2nd
	VEHSDLSFS[K]	passed	passed	NA
OPN	ISHELDSASSE[V]N	passed	passed	NA
	GDSVVYGL[R]	passed	passed	1st; 2nd
BSA	DLGEEHF[K]	passed	passed	NA
	HLVDEPQNLI[K]	passed	passed	NA
	LGEYGFQNALIV[R]	passed	passed	1st; 2nd

The isotope labelled residues are shown beteen brackets (modified Lysine [K]: + 8 Da , modified Arginine [R]: + 10 Da, modified Valine [V]: + 6 Da)

NA: Not Applicable

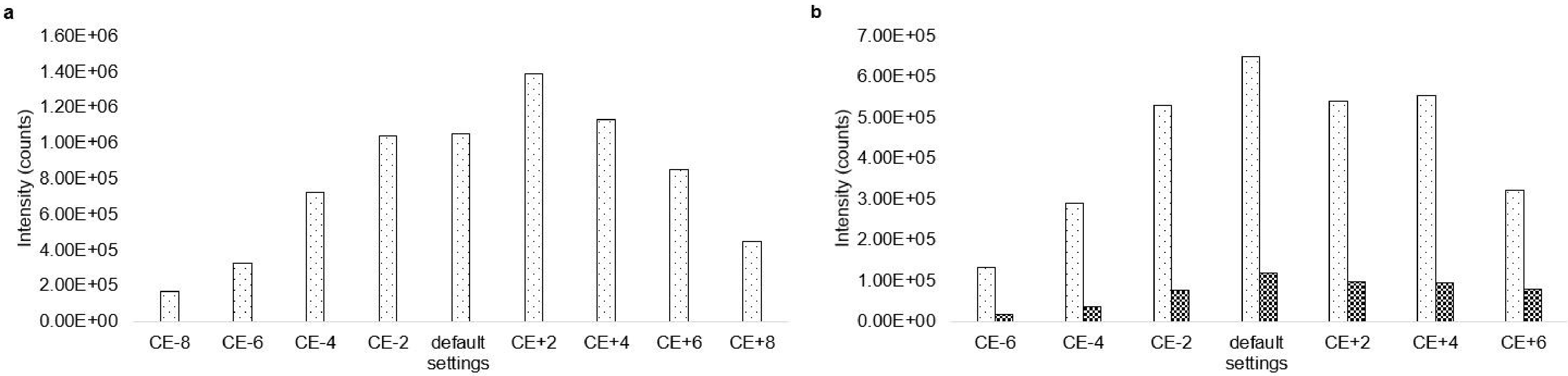

Figure S2. Optimization of collision energy (CE) for CC16 and RBP4. Comparison of the intensity (counts) obtained by varying stepwise the CE values for the transitions pep3_1 (▫) of RBP4 (a) and for the transitions pep3_1 (▫) and pep3_3 (▪) of CC16 (b)

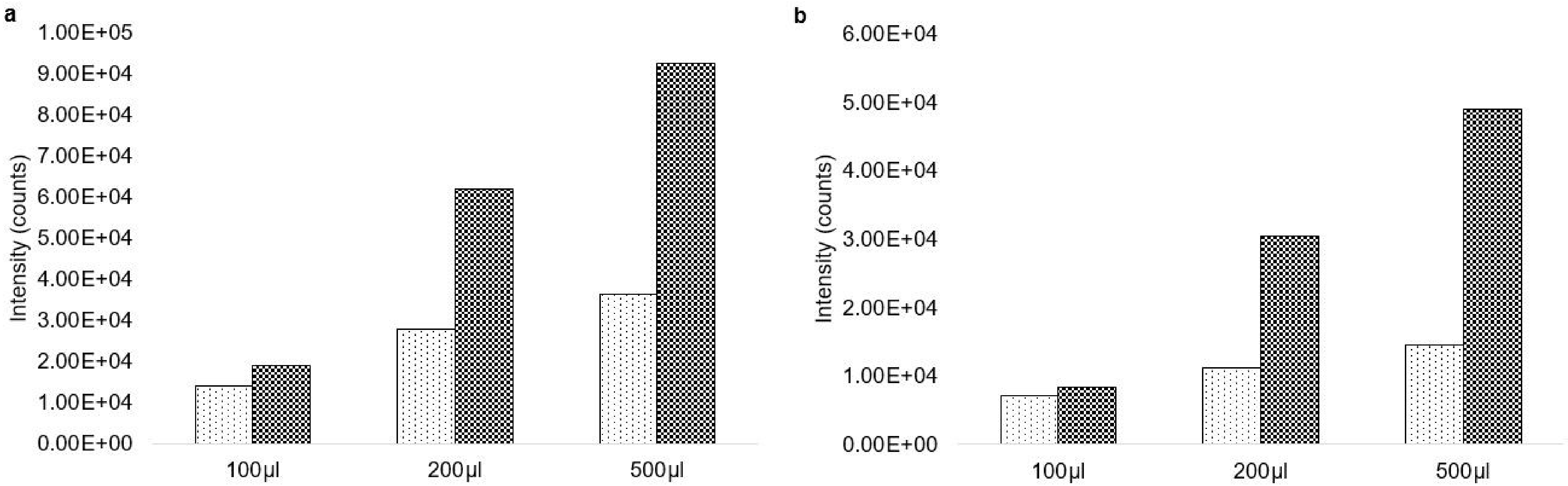

Figure S3. Trypsin digest on varying volumes of urine (100 µl, 200 µl, 500 µl). Intensity (counts) of the labelled peptide transitions CC16 pep3_1 (a) and pep3_3 (b) in urine spiked with 100ng/ml (▫) or 200 ng/ml (▪) of CC16 protein.

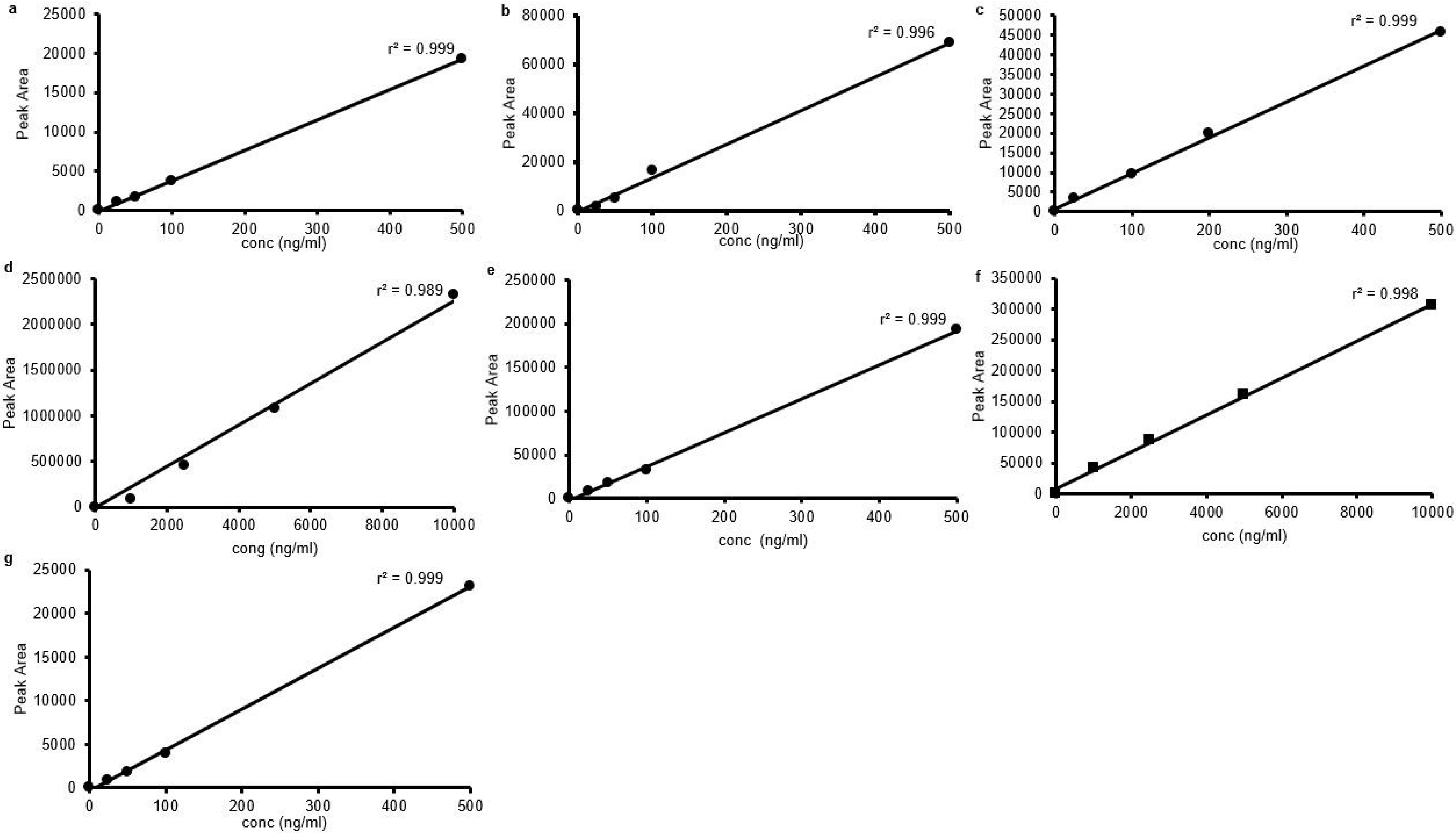

Figure S4. Standard curves of CC16 (a) , β2M (b), RBP4 (c), OPN (d), NF-κB (e), HSA (f) and MYO (g)

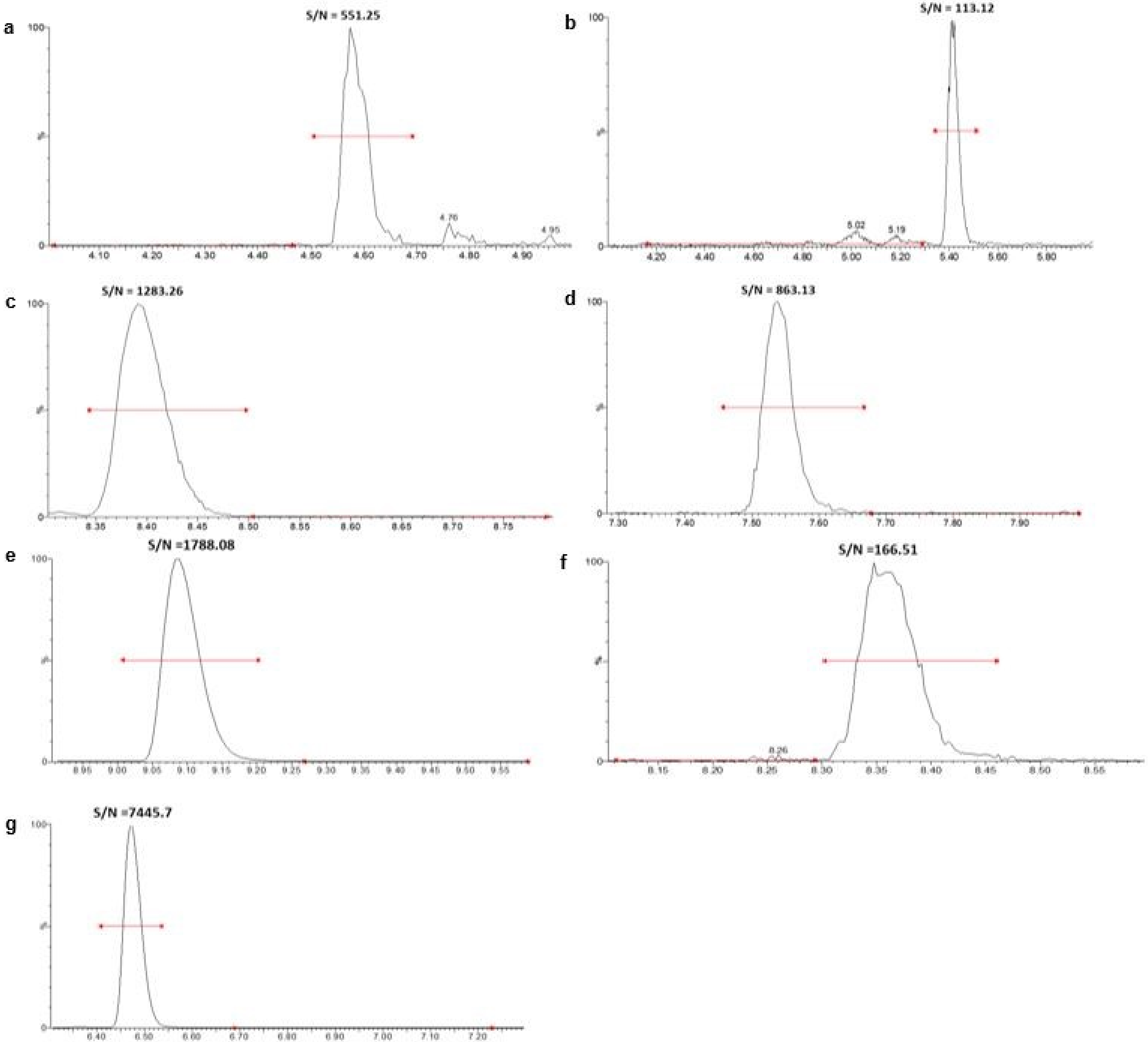

Figure S5. Signal-to-noise (S/N) ratio of CC16 (a), β2M (b), RBP4 (c), NF-κB (d), HSA (e), MYO (f), OPN (g)

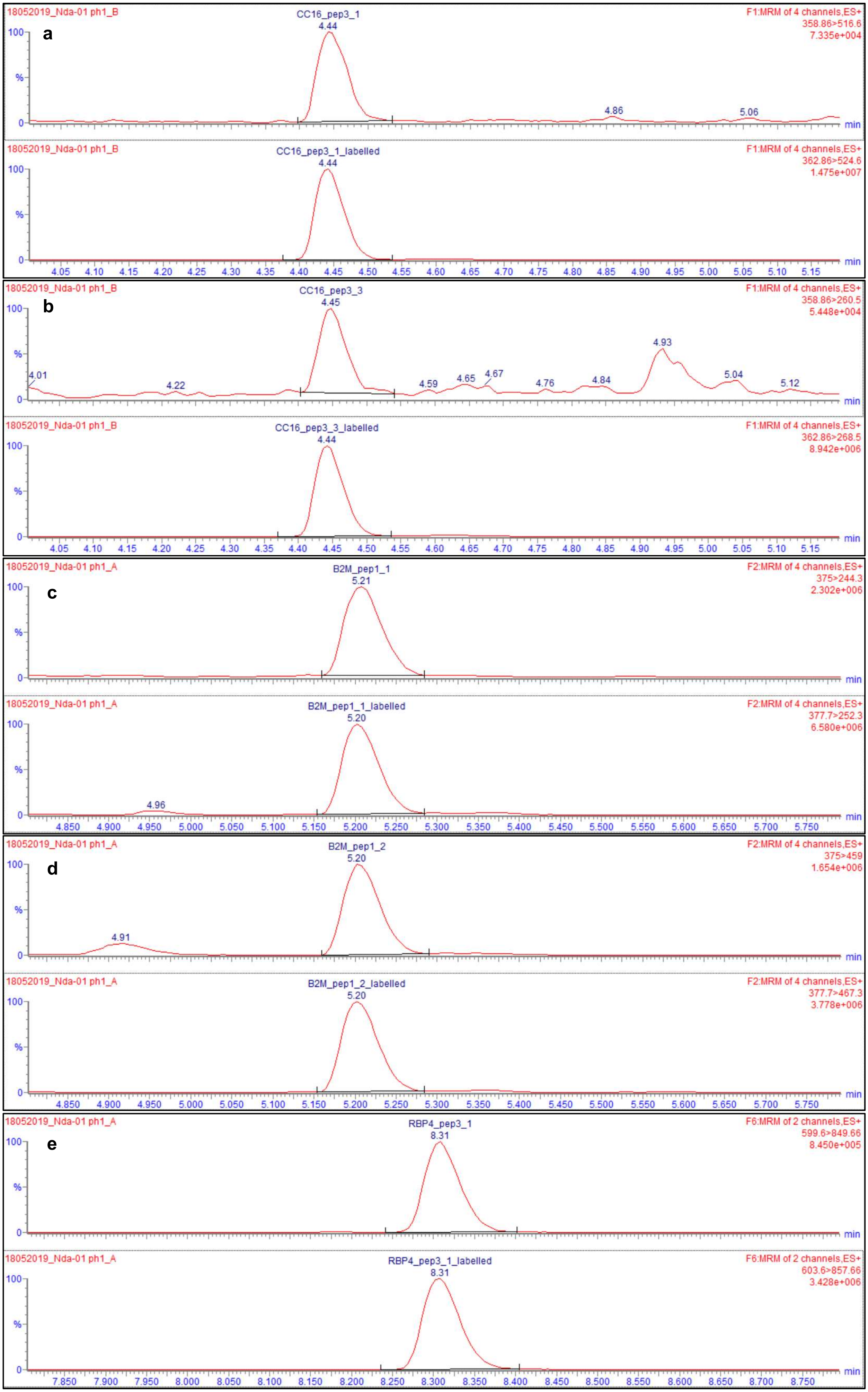
a
18052019_Nda-01 ph1_B
CC16_pep3_1
4.44
F1:MRM of 4 channels,ES+
358.86>516.6
7.335e+004
4.86
5.06
18052019_Nda-01 ph1_B
CC16_pep3_1_labelled
4.44
F1:MRM of 4 channels,ES+
362.86>524.6
1.475e+007
b
18052019_Nda-01 ph1_B
CC16_pep3_3
4.45
F1:MRM of 4 channels,ES+
358.86>260.5
5.448e+004
4.01
4.22
4.59
4.65
4.67
4.76
4.84
4.93
5.04
5.12
18052019_Nda-01 ph1_B
CC16_pep3_3_labelled
4.44
F1:MRM of 4 channels,ES+
362.86>268.5
8.942e+006
c
18052019_Nda-01 ph1_A
B2M_pep1_1
5.21
F2:MRM of 4 channels,ES+
375>244.3
2.302e+006
18052019_Nda-01 ph1_A
B2M_pep1_1_labelled
5.20
F2:MRM of 4 channels,ES+
377.7>252.3
6.580e+006
4.96
d
18052019_Nda-01 ph1_A
B2M_pep1_2
5.20
F2:MRM of 4 channels,ES+
375>459
1.654e+006
4.91
18052019_Nda-01 ph1_A
B2M_pep1_2_labelled
5.20
F2:MRM of 4 channels,ES+
377.7>467.3
3.778e+006
e
18052019_Nda-01 ph1_A
RBP4_pep3_1
8.31
F6:MRM of 2 channels,ES+
599.6>849.66
8.450e+005
18052019_Nda-01 ph1_A
RBP4_pep3_1_labelled
8.31
F6:MRM of 2 channels,ES+
603.6>857.66
3.428e+006
100
%
0
min

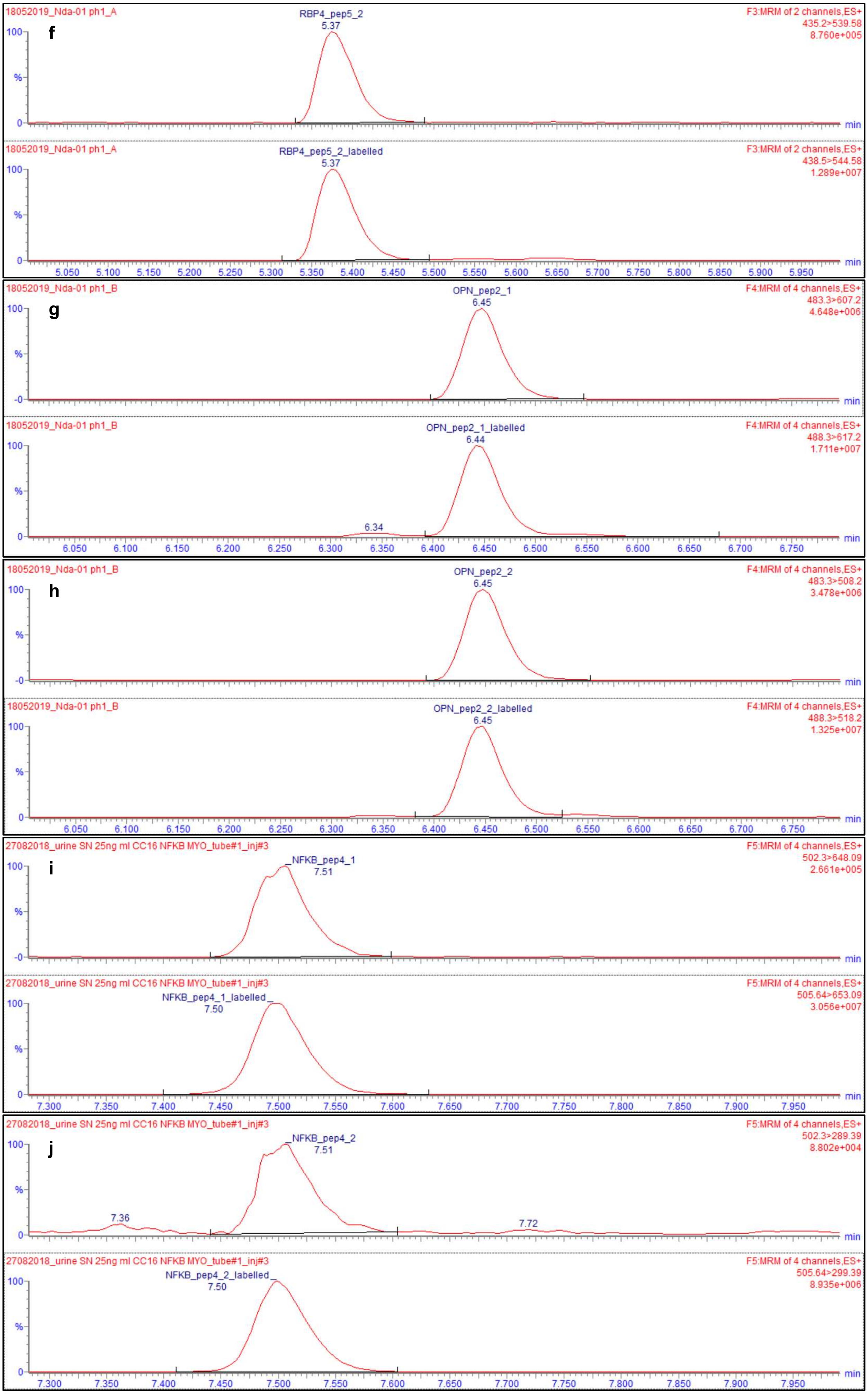

f
18052019_Nda-01 ph1_A
RBP4_pep5_2
5.37
F3:MRM of 2 channels,ES+
435.2>539.58
8.760e+005
18052019_Nda-01 ph1_A
RBP4_pep5_2_labelled
5.37
F3:MRM of 2 channels,ES+
438.5>544.58
1.289e+007
g
18052019_Nda-01 ph1_B
OPN_pep2_1
6.45
F4:MRM of 4 channels,ES+
483.3>607.2
4.648e+006
18052019_Nda-01 ph1_B
OPN_pep2_1_labelled
6.44
6.34
F4:MRM of 4 channels,ES+
488.3>617.2
1.711e+007
h
18052019_Nda-01 ph1_B
OPN_pep2_2
6.45
F4:MRM of 4 channels,ES+
483.3>508.2
3.478e+006
18052019_Nda-01 ph1_B
OPN_pep2_2_labelled
6.45
F4:MRM of 4 channels,ES+
488.3>518.2
1.325e+007
i
27082018_urine SN 25ng ml CC16 NFKB MYO_tube#1_inj#3
NFKB_pep4_1
7.51
F5:MRM of 4 channels,ES+
502.3>648.09
2.661e+005
27082018_urine SN 25ng ml CC16 NFKB MYO_tube#1_inj#3
NFKB_pep4_1_labelled
7.50
F5:MRM of 4 channels,ES+
505.64>653.09
3.056e+007
j
27082018_urine SN 25ng ml CC16 NFKB MYO_tube#1_inj#3
NFKB_pep4_2
7.51
7.36
7.72
F5:MRM of 4 channels,ES+
502.3>289.39
8.802e+004
27082018_urine SN 25ng ml CC16 NFKB MYO_tube#1_inj#3
NFKB_pep4_2_labelled
7.50
F5:MRM of 4 channels,ES+
505.64>299.39
8.935e+006
min
%

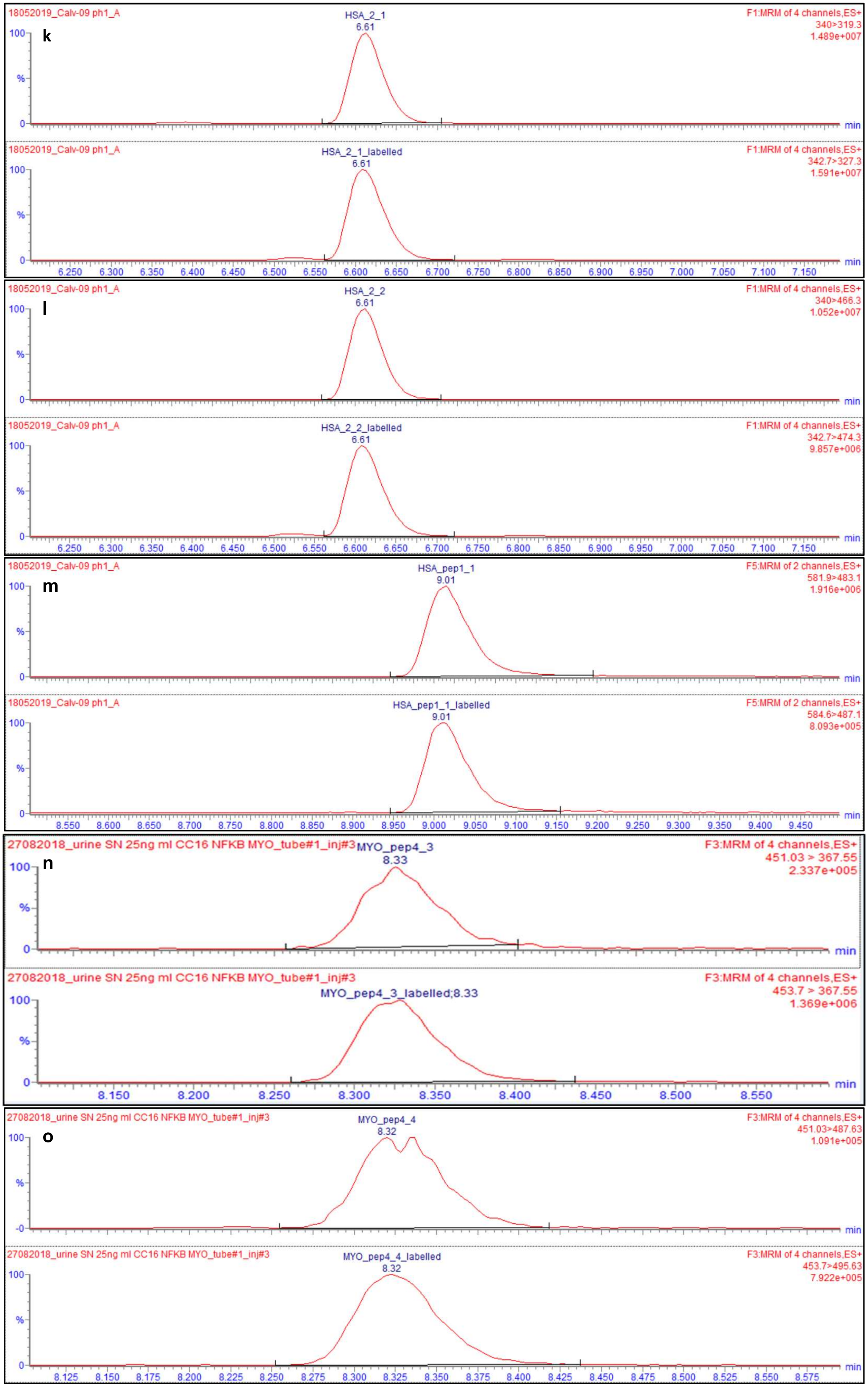
k
18052019_Calv-09 ph1_A
HSA_2_1
6.61
F1:MRM of 4 channels,ES+
340>319.3
1.489e+007
18052019_Calv-09 ph1_A
HSA_2_1_labelled
6.61
F1:MRM of 4 channels,ES+
342.7>327.3
1.591e+007
l
18052019_Calv-09 ph1_A
HSA_2_2
6.61
F1:MRM of 4 channels,ES+
340>466.3
1.052e+007
18052019_Calv-09 ph1_A
HSA_2_2_labelled
6.61
F1:MRM of 4 channels,ES+
342.7>474.3
9.857e+006
m
18052019_Calv-09 ph1_A
HSA_pep1_1
9.01
F5:MRM of 2 channels,ES+
581.9>483.1
1.916e+006
18052019_Calv-09 ph1_A
HSA_pep1_1_labelled
9.01
F5:MRM of 2 channels,ES+
584.6>487.1
8.093e+005
n
27082018_urine SN 25ng ml CC16 NFKB MYO_tube#1_inj#3
MYO_pep4_3
8.33
F3:MRM of 4 channels,ES+
451.03 > 367.55
2.337e+005
27082018_urine SN 25ng ml CC16 NFKB MYO_tube#1_inj#3
MYO_pep4_3_labelled;8.33
F3:MRM of 4 channels,ES+
453.7 > 367.55
1.369e+006
o
27082018_urine SN 25ng ml CC16 NFKB MYO_tube#1_inj#3
MYO_pep4_4
8.32
F3:MRM of 4 channels,ES+
451.03>487.63
1.091e+005
27082018_urine SN 25ng ml CC16 NFKB MYO_tube#1_inj#3
MYO_pep4_4_labelled
8.32
F3:MRM of 4 channels,ES+
453.7>495.63
7.922e+005
min

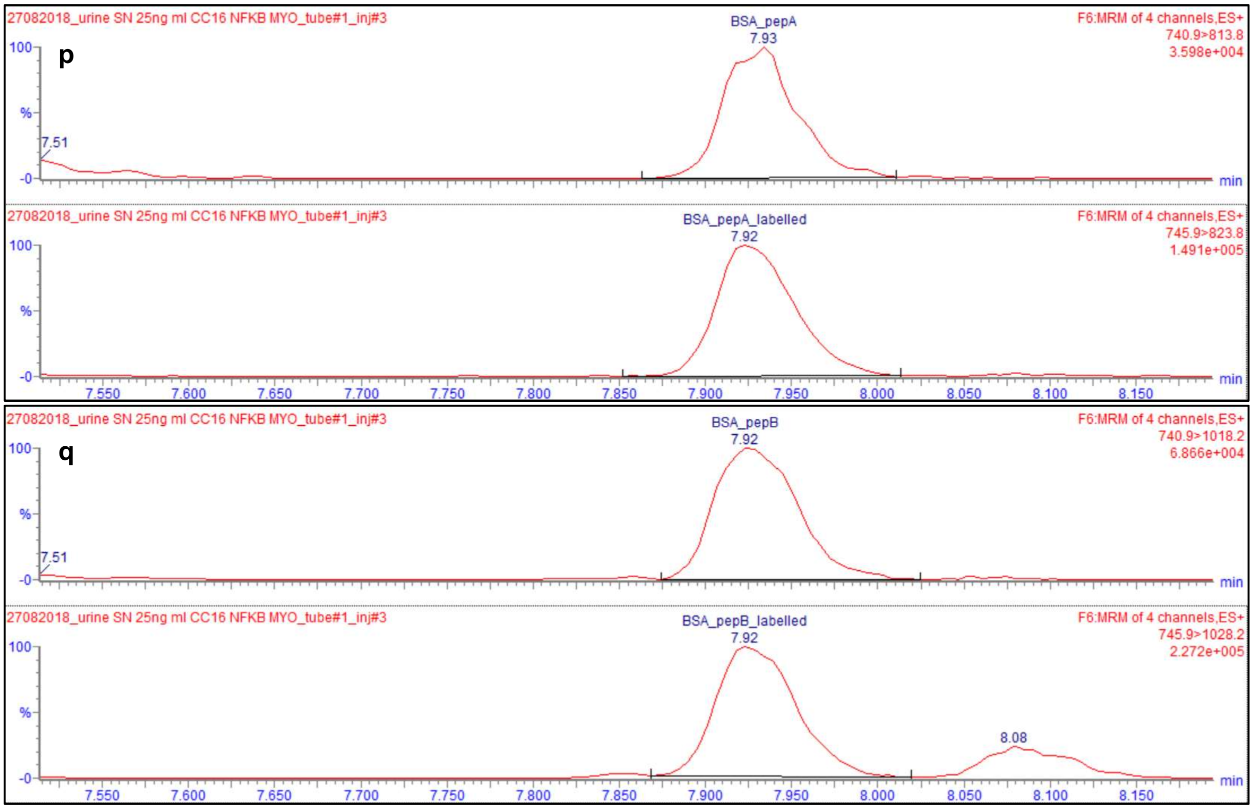

Figure S6. Typical chromatograms of coelution of native and isotope labelled proteotypic peptides from urine samples : CC16 pep3_1 (a), CC16 pep3_3 (b), B2M pep1_1 (c), B2M pep1_2 (d), RBP4 pep3_1 (e), RBP4 pep5_2 (f), OPN pep2_1 (g), OPN pep2_2 (h), NFKB pep4_1 (i), NFKB pep4_2 (j), HSA pep2_1 (k), HSA pep2_2 (l), HSA pep1_1 (m), MYO pep4_3 (n), MYO pep4_4 (o), BSA pep3_A (p), BSA pep3_B (q)

Table S7. Transition list with the following information: Identifier for targeted protein (protein_name), peptide sequence inluding any modification (sequence), unique identification for the transition of certain precursor (transition_group_id), which labeling used, heavy or light (isotype), precursor m/z setting for quadrupole 1 (Q1) and quadrupole 3 (Q3), time spent on transition (ms) (dwelltime), retention time for the scheduled SRM measurement (minutes) (Tr_recalibrated), collusion energy used for this precursor (CE), precursor charge state (prec_z), fragment ion type (frg_type), fragment number (frg_nr), fragment charge state (frg_z)

protein_name	sequence	transition_group_id	isotype	Q1	Q3	dwelltime	Tr_recalibrated	CE	prec_z	frg_type	frg_nr	frg_z
CC16	EAGAQLK	CC16_pep3_1	light	358.9	516.6	0.08	4.5	10	2	y	5	1
CC16	EAGAQLK[8]	CC16_pep3_1_labelled	heavy	362.9	524.6	0.08	4.5	10	2	y	5	1
CC16	EAGAQLK	CC16_pep3_3	light	358.9	260.5	0.08	4.5	7	2	y	2	1
CC16	EAGAQLK[8]	CC16_pep3_3_labelled	heavy	362.9	268.5	0.08	4.5	7	2	y	2	1
RBP4	YWGVASFLQK	RBP4_pep3_1	light	599.6	849.7	0.052	8.4	9	2	y	8	1
RBP4	YWGVASFLQK[8]	RBP4_pep3_1_labelled	heavy	603.6	857.7	0.052	8.4	9	2	y	8	1
RBP4	LIVHNGYCDGR	RBP4_pep5	light	435.2	539.6	0.051	5.5	9	3	y	9	2
RBP4	LIVHNGYCDGR[10]	RBP4_pep5_labelled	heavy	438.5	544.6	0.051	5.5	9	3	y	9	2
B2M	VNHVTLSQPK	B2M_pep1_1	light	375.0	244.3	0.029	5.3	20	3	y	2	1
B2M	VNHVTLSQPK[8]	B2M_pep1_1_labelled	heavy	377.7	252.3	0.029	5.3	20	3	y	2	1
B2M	VNHVTLSQPK	B2M_pep1_2	light	375.0	459.0	0.029	5.3	12	3	y	4	1
B2M	VNHVTLSQPK[8]	B2M_pep1_2_labelled	heavy	377.7	467.3	0.029	5.3	12	3	y	4	1
OPN	GDSVVYGLR	OPN_2_1	light	483.3	607.2	0.038	6.4	15	2	y	5	1
OPN	GDSVVYGLR[10]	OPN_2_1_labelled	heavy	488.3	617.2	0.038	6.4	15	2	y	5	1
OPN	GDSVVYGLR	OPN_2_2	light	483.3	508.2	0.038	6.4	15	2	y	4	1
OPN	GDSVVYGLR[10]	OPN_2_2_labelled	heavy	488.3	518.2	0.038	6.4	15	2	y	4	1
HSA	SLHTLFGDK	HSA_2_1	light	340.0	319.3	0.037	6.7	12	3	y	3	1
HSA	SLHTLFGDK[8]	HSA_2_1_labelled	heavy	342.7	327.3	0.037	6.7	12	3	y	3	1
HSA	SLHTLFGDK	HSA_2_2	light	340.0	466.3	0.037	6.7	12	3	y	4	1
HSA	SLHTLFGDK[8]	HSA_2_2_labelled	heavy	342.7	474.3	0.037	6.7	12	3	y	4	1
HSA	HPYFYAPELLFFAK	HSA_1_1	light	581.9	483.1	0.163	9.0	15	3	y	8	2
HSA	HPYFYAPELLFFAK[8]	HSA_1_1_labelled	heavy	584.6	487.1	0.163	9.0	15	3	y	8	2
MYO	HGATVLTALGGILK	MYO_pep4_3	light	451.0	367.6	0.038	8.2	10	3	b	4	1
MYO	HGATVLTALGGILK[8]	MYO_pep4_3_labelled	heavy	453.7	367.6	0.038	8.2	10	3	b	4	1
MYO	HGATVLTALGGILK	MYO_pep4_4	light	451.0	487.6	0.038	8.2	16	3	y	5	1
MYO	HGATVLTALGGILK[8]	MYO_pep4_4_labelled	heavy	453.7	495.6	0.038	8.2	16	3	y	5	1
NF-KB	LPPVLSHPIFDNR	NFKB_pep4_1	light	502.3	648.1	0.038	7.4	12	3	y	11	2
NF-KB	LPPVLSHPIFDNR[10]	NFKB_pep4_1_labelled	heavy	505.6	653.1	0.038	7.4	12	3	y	11	2
NF-KB	LPPVLSHPIFDNR	NFKB_pep4_2	light	502.3	289.4	0.038	7.4	20	3	y	2	1
NF-KB	LPPVLSHPIFDNR[10]	NFKB_pep4_2_labelled	heavy	505.6	299.4	0.038	7.4	20	3	y	2	1
BSA	LGEYGFQNALIVR	BSA_pep3_A	light	740.9	813.8	0.024	7.9	30	2	y	7	1
BSA	LGEYGFQNALIVR[10]	BSA_pep3_A_labelled	heavy	745.9	823.8	0.024	7.9	30	2	y	7	1
BSA	LGEYGFQNALIVR	BSA_pep3_B	light	740.9	1018.2	0.024	7.9	30	2	y	9	1
BSA	LGEYGFQNALIVR[10]	BSA_pep3_B_labelled	heavy	745.9	1028.2	0.024	7.9	30	2	y	9	1
